# Molecular characterization and epidemiology of *Streptococcus pneumoniae* serotype 8 in Denmark

**DOI:** 10.1186/s12879-021-06103-w

**Published:** 2021-05-05

**Authors:** Camilla Bülow Hansen, Kurt Fuursted, Palle Valentiner-Branth, Tine Dalby, Charlotte Sværke Jørgensen, H-C Slotved

**Affiliations:** 1 Department of Bacteria, Parasites and Fungi, Statens Serum Institut, Artillerivej 5, DK-2300 Copenhagen S, Denmark; 2 Infectious Disease Epidemiology and Prevention, Statens Serum Institut, Copenhagen, Denmark; 3 Department of Virus and Microbiological Special Diagnostics, Statens Serum Institut, Copenhagen, Denmark

**Keywords:** Denmark, Whole genome sequencing, MLST, Serotype 8, IPD, PCV13, *Streptococcus pneumoniae*

## Abstract

**Background:**

*Streptococcus pneumoniae* serotype 8 incidence has increased in Denmark after the introduction of pneumococcal conjugated vaccines (PCV). The mechanism behind the serotype 8 replacement is not well understood. In this study, we aimed to present epidemiological data on invasive pneumococcal disease (IPD) and molecular characterization of 96 serotype 8 clinical isolates.

**Methods:**

IPD data from 1999 to 2019 were used to calculate the incidence and age distribution. Whole-genome sequencing (WGS) analysis was performed on 96 isolates (6.8% of the total serotype 8 IPD isolates in the period) to characterize the isolates with respect to pneumococcal lineage traits, a range of genes with potential species discrimination, presence of colonization and virulence factors, and molecular resistance pattern.

**Results:**

The serotype 8 IPD incidence increased significantly (*P* < 0.05) for the age groups above 15 years after the introduction of PCV13, primarily affecting the elderly (65+). All isolates were phenotypically susceptible to penicillin, erythromycin and clindamycin.

Molecular characterization revealed seven different MLST profiles with ST53 as the most prevalent lineage (87.5%) among the analyzed serotype 8 isolates. The genes covering the cell-surface proteins: *lytA*, *rspB*, *pspA*, *psaA* & *Xisco* and the pneumococcal toxin pneumolysin = *ply* were present in all isolates, while genes for the membrane transporter proteins: *piaA/piaB/piaC*; the capsular genes: *cpsA (wzg)* & *psrP*; the metallo-binding proteins *zmpB* & *zmpC*; and the neuroamidase proteins: *nanA/nanB* were variably present. Surprisingly, the putative transcriptional regulator gene *SP2020* was not present in all isolates (98%). Susceptibility to penicillin, erythromycin and clindamycin was molecularly confirmed.

**Conclusion:**

The observed serotype 8 replacement was not significantly reflected with a change in the MLST profile or changes in antibiotic resistance- or virulence determinants.

**Supplementary Information:**

The online version contains supplementary material available at 10.1186/s12879-021-06103-w.

## Background

Infections with *Streptococcus pneumoniae* affect all age groups, although predominantly children and the elderly. Invasive pneumococcal diseases (IPD) such as bacteremia, meningitis, and pneumonia cause high morbidity and mortality worldwide [1] and can be divided into serotypes based on their capsular polysaccharide with up to least 100 acknowledged serotypes [2, 3].

The introduction of the pneumococcal conjugate vaccine (PCV) – 7-valent (PCV7) in 2007 and 13-valent (PCV13) in 2010 – has changed the epidemiology of serotype-specific IPD in Denmark [4]. While the IPD incidence for the majority of serotypes included in the PCV13 vaccine has decreased, the incidence of non-PCV serotypes such as serotype 8 has increased post PCV vaccination [5]. The frequency of serotype 8 IPD has increased globally from 2013 to 2017 with 120% [6]. The observed replacement and increase of serotype 8 is similar to the emergence of the virulent and multidrug resistant serotype 19A observed in Massachusetts, USA, after introduction of PCV7, although an increase in multidrug resistance has not been observed [7].

In Europe, the current serotype 8 multi locus sequence type (MLST) is dominated by ST53 [8, 9], which constitutes up to 90.1% in Spain [9], and is related to the major clone Netherlands^8^–33 (https://www.pneumogen.net/pmen/clone-collection.html, accessed 14–04–2021) [10]. Alteration in the clonal epidemiology or other virulence related pneumococcal genes could be the explanation for the post PCV increase of serotype 8, as observed with serotype 19A ST320 post PCV7 in USA [7] or with serotype 19A ST199 and ST994 and the change in PCV strategies in Belgium [11]. In Denmark, we have observed a post PCV13 increase in serotype 24F ST162 [12]. However, even though the non-PCV serotype 8 for several years has been the dominant cause of IPD in Denmark, little is known about the serotype.

Serotype 8 is an example of successful serotype replacement due to PCV introduction in Denmark. It is, therefore, the intention of this study to investigate the mechanism behind the significant increase in serotype 8 IPD cases post PCV, by evaluating epidemiological data, the clonal epidemiology, and the presence/absence of virulence related pneumococcal genes. Whole-genome sequencing (WGS) analysis was performed on a representative number of serotype 8 isolates to investigate potential changes in the clonal distribution, molecularly susceptible related genes, and species-specific virulence genes pre- and post- PCV vaccination.

## Methods

### Strain collection

Data from all *S. pneumoniae* serotype 8 IPD isolates from 1999 to 2019 were retrieved from the national Neisseria and Streptococcus Reference Laboratory (NSR), Statens Serum Institut (SSI) (Table 1). The registered *S. pneumoniae* serotype 8 IPD cases where either isolates or pneumococcal DNA obtained from sterile fluids such as blood, cerebrospinal fluid, joint fluid etc. [13]. The cases consisted of more than 90% bacteremia cases, 5% meningitis cases, and less than 5% other infections found in normally sterile sites. Due to the limited number of meningitis cases and other infections these data were combined in one group. Detailed data on the total number of IPD cases in Denmark for all age groups have previously been presented [4, 5]. Population data (1999–2019) were obtained from Statistics Denmark (www.dst.dk, accessed 10–03-2021). The number of specific serotype 8 IPD cases and of total IPD cases per year in Denmark is presented in Table 1.

**Table 1:** The number of serotype 8 IPD cases per age group from 1999 to 2019

	The number of serotype 8 IPD isolates/and total number of IPD from 1999 to 2019 per age group (% of total)								
Year	< 2	2–4	5–14	15–44	45–64	65–74	75–84	85+	All age groups
1999	2/54 (3.7%)	0/13 (0%)	1/17 (5.9%)	8/90 (8.9%)	13/215 (6.0%)	14/210 (6.7%)	10/187 (5.3%)	2/60 (3.3)	50/846 (5.9%)
2000	0/50 (0%)	0/12 (0%)	0/7 (0%)	7/86 (8.1%)	15/234 (6.4%)	10/167 (6.0%)	7/166 (4.2%)	2/97 (2.1%)	41/819 (5.0%)
2001	1/62 (1.6%)	0/19 (0%)	0/12 (0%)	5/109 (4.6%)	23/295 (7.8%)	12/195 (6.2%)	6/183 (0.33%)	4/105 (3.8%)	51/980 (5.2%)
2002	0/68 (0%)	0/19 (0%)	1/18 (5.6%)	7/155 (4.5%)	18/287 (6.3%)	17/213 (8.0%)	14/228 (6.2%)	2/100 (2.0%)	61/1088 (5.6%)
2003	1/70 (1.4%)	0/22 (0%)	1/31 (3.2)	11/156 (7.1)	19/351 (5.4%)	15/223 (6.7%)	13/253 (5.1%)	4/114 (3.5%)	64/1220 (5.2%)
2004	1/76 (1.3%)	0/21 (0%)	4/29 (13.8)	9/155 (5.8%)	12/302 (4.0%)	13/234 (5.6%)	11/280 (3.9%)	3/115 (2.6%)	54/1212 (4.5%)
2005	0/68 (0%)	0/14 (0%)	0/23 (0%)	9/114 (7.9%)	20/305 (6.6%)	13/226 (5.8%)	13/225 (5.8%)	5/119 (4.2%)	60/1094 (5.5%)
2006	1/83 (1.2%)	0/13 (0%)	0/18 (0%9	8/84 (9.5%)	20/257 (7.8%)	13/176 (7.4%)	6/175 (3.4%)	3/98 (3.1%)	51/904 (5.6%)
2007	0/97 (0%)	0/16 (0%)	0/16 (0%)	7/129 (5.4%)	22/294 (7.5%)	8/205 (3.9%)	10/244 (4.1%)	3/114 (2.6%)	50/1115 (4.5%)
In 2007, the PCV7 was introduced in Denmark									
2008	0/32 (0%)	0/23 (0%)	0/12 (0%)	9/98 (9.2%)	24/263 (9.1%)	13/223 (5.8%)	12/185 (6.5%)	10/125 (8.0%)	68/961 (7.1%)
2009	1/42 (2.4%)	0/27 (0%)	0/15 (0%)	6/128 (4.7%)	19/294 (6.5%)	11/228 (4.8%)	19/210 (9.0%)	8/123 (6.5%)	64/1067 (6.0%)
2010	1/33 (3.0)	0/12 (0%)	0/16 (0%)	6/130 (4.6%)	19/308 (6.2%)	18/254 (7.1%)	12/210 (5.7%)	2/95 (2.1%)	58/1058 (5.5%)
In 2010, the PCV13 was introduced in Denmark									
2011	1/17 (5.9%)	0/17 (0%)	0/26 (0%)	3/98 (3.1%)	23/283 (8.1%)	17/240 (7.1%)	17/195 (8.7%)	4/118 (3.4%)	65/994 (6.5%)
2012	0/23 (0%)	0/15 (0%)	0/20 (0%)	7/89 (7.9%)	24/272 (8.8%)	15/233 (6.4%)	10/207 (4.8%)	8/103 (7.8%)	64/962 (6.7%)
2013	1/21 (4.8%)	0/10 (0%)	2/21 (9.5%)	9/97 (9.3%)	39/263 (14.8%)	22/201 (10.9%)	18/165 (10.9%)	11/112 (9.8%)	102/890 (11.5%)
2014	1/29 (3.4%)	0/10 (0%)	1/7 (14.3%)	11/57 (19.3%)	40/196 (20.4%)	35/219 (16.0%)	27/165 (10.3%)	12/93 (12.9%)	127/776 (16.4%)
2015	2/15 (13.3%	0/5 (0%)	1/7 (14.3%)	23/73 (31.5%)	33/188 (17.6%)	60/243 (24.7%)	34/165 (20.6%)	12/111 (10.8%)	165/807 (20.4%)
2016	4/16 (24%)	0/5 (0%)	1/4 (24%)	18/61 (29.5%)	50/187 (26.7%)	53/213 (24.9%	30/158 (19.0%)	14/87 (16.1%)	170/731 (23.3%)
2017	2/21 (9.5%)	0/7 (0%)	2/10 (20%)	20/50 (40%)	62/209 (29.7%)	58/216 (26.9%)	34/154 (22.1%)	19/105 (18.1%)	198/772 (25.5%)
2018	1/13 (7.7%)	0/4 (0%)	0/9 (0%)	25/53 (47.2%)	56/193 (29.0%)	59/237 (24.9%)	30/171 (17.5%)	26/119 (21.8%)	197/799 (24.7%)
2019	1/14 (7.1%	0/2 (0%)	0/1 (0%)	18/43 (41.9%)	42/164 25.6%)	47/180 (26.1%)	26/135 (19.3%)	16/90 (17.8%)	150/639 (23.5%)

### Data analysis

The data were analyzed using RStudio version 1.2.5001 and R version R-3.6.1 (http://www.r-project.org/, accessed 10–03-2021) for descriptive statistical analysis. Calculations consisted of t-test, two-tailed Fisher’s exact test, and a generalized linear model to calculate incidence rate, incidence rate ratio (IRR), *p*-value, and confidence interval (CI).

### Identification of *S. pneumoniae* isolates

The serotype 8 IPD isolates were phenotypically species-confirmed based on optochin susceptibility and identification of the serotype. Serotyping was performed either by the Quellung reaction alone or by the ImmuLex™ Pneumotest Kit (SSIDiagnostica.com, Hillerød, Denmark) combined with the Quellung reaction using type-specific pneumococcal rabbit-antisera (SSIDiagnostica.com, Hillerød, Denmark) [12, 14].

### Characterization of 96 clinically selected isolates

From a total of 1378 *S. pneumoniae* serotype 8 IPD isolates collected from 2006 to 2018, 92 (6%) were selected for molecular characterization using WGS. Of the selected isolates, two were from 2006 and 10 isolates (90 isolates in total) were selected from each of the years 2007, 2009–2014, 2016, and 2018. The isolates were not randomly selected but chosen based on the 65+ age group, gender, and collection site, and represented patients from all parts of the country. The isolates were from patients with a mean age of 72.7 years, 50% of the isolates were from female patients, and 90% were bacteremic cases.

Additionally, 4 historical serotype 8 isolates were included, two isolates (Serotype 8 (Lederle) strain (nb 27, isolate number 8-2-1950) and serotype 8 Henrique strain (nb 28), isolate number 8-1-1950) both from the Lederle laboratories, Pearl River, USA [15] with unknown gender and site of infection and two serotype 8 isolates from Denmark (1962) from cerebrospinal fluid samples (isolates 8-3-1962 and 8-4-1962).

Data on the 96 selected isolates are presented in Table 2.

**Table 2:** Selected genes detected in the *S. pneumoniae* isolates. These parameters were used for positive gene detection: *Cut-off of overlap as 80 and 95% identity. LytA* (HG531769.1), *ply* (KP110598), *Xisco* (*SP_1992*) (AAK76059.1), *rpsB* (from TIGR4 AE005672.3: 2,134,264–2,135,043)*, pspA* (AF516671), and *psaA* (U53509.1) were detected in all 96 isolates. *cpsA (wzg)* (AF057294:2134–2473), *zmpB* (AAK74809.1, and *psrP* (CS819261.1) were not detected in any of the isolates. The distribution of the genes *piaA*/*piaB*/*piaC* (AF338658.1), *zmpC* (AAK74260.1), *nanA*/*nanB* (U43526.1), and *SP2020* (AAK76085.1) are presented below. The genetic antimicrobial profile of the isolates is presented below. All isolates were penicillin sensitive. The isolates were not tested for tetracycline

Reference	Year	Focus site	MLST	GPSC	PBP profile	Resistance genes	*piaA*	*piaB*	*piaC*	*zmpC*	*nanA*/*nanB*	*SP2020*
8–1	1950	Unknown	ST53	3	3–6-5	None	Yes	Yes	Yes	Yes	Yes	Yes
8–2	1950	Unknown	ST53	3	3–6-5	None	Yes	Yes	Yes	Yes	Yes	Yes
8–3	1962	Cerebrospinal fluid	ST53	3	3–6-5	None	Yes	Yes	Yes	Yes	Yes	Yes
8–4	1962	Cerebrospinal fluid	ST7203	98	3–2-5	None	Yes	Yes	Yes	No	No	Yes
Number of isolates	Year	Focus site	MLST	GPSC	PBP profile	Resistance genes	*piaA*	*piaB*	*piaC*	*zmpC*	*nanA*/*nanB*	*SP2020*
1	2006	Cerebrospinal fluid	ST53	3	3–6-5	tet(M)	Yes	Yes	Yes	Yes	Yes	Yes
1	2006	Cerebrospinal fluid	ST404	98	3–2-5	None	Yes	Yes	Yes	No	No	Yes
In 2007 was the PCV7 introduced in Denmark												
1	2007	Blood	ST404	98	3–2-5	None	Yes	Yes	Yes	No	Yes	Yes
1	2007	Blood	ST404	98	3–2-5	None	Yes	Yes	Yes	No	No	Yes
5	2007	Blood	ST53	3	3–6-5	None	Yes	Yes	Yes	Yes	Yes	Yes
1	2007	Cerebrospinal fluid	ST404	98	3–2-5	None	Yes	Yes	Yes	No	No	Yes
1	2007	Blood	ST1480	98	3–2-5	None	Yes	Yes	Yes	No	No	Yes
1	2007	Blood	ST53	3	3–6-5	tet(M)	Yes	Yes	Yes	Yes	Yes	Yes
8	2009	Blood	ST53	3	3–6-5	None	Yes	Yes	Yes	Yes	Yes	Yes
1	2009	Cerebrospinal fluid	ST53	3	3–6-5	None	Yes	Yes	Yes	Yes	Yes	Yes
1	2009	Blood	ST3714	224	50–0-0	None	Yes	Yes	Yes	No	Yes	Yes
In 2010 was the PCV13 introduced in Denmark												
8	2010	Blood	ST53	3	3–6-5	None	Yes	Yes	Yes	Yes	Yes	Yes
1	2010	Blood	New ST type	3	3–6-5	tet(M)	Yes	Yes	Yes	Yes	Yes	Yes
1	2010	Blood	ST1480	98	3–2-5	None	Yes	Yes	Yes	No	No	Yes
4	2011	Cerebrospinal fluid	ST53	3	3–6-5	None	Yes	Yes	Yes	Yes	Yes	Yes
1	2011	Blood	ST53	3	3–6-5	None	Yes	No	No	Yes	Yes	Yes
5	2011	Blood	ST53	3	3–6-5	None	Yes	Yes	Yes	Yes	Yes	Yes
8	2012	Blood	ST53	3	3–6-5	None	Yes	Yes	Yes	Yes	Yes	Yes
1	2012	Cerebrospinal fluid	ST53	3	3–6-5	None	Yes	Yes	Yes	Yes	Yes	Yes
1	2012	Blood	ST2234	336	0–4-2	None	Yes	Yes	Yes	No	Yes	Yes
8	2013	Blood	ST53	3	3–6-5	None	Yes	Yes	Yes	Yes	Yes	Yes
1	2013	Blood	ST53	3	3–6-5	None	No	Yes	Yes	Yes	Yes	Yes
1	2013	Blood	ST404	98	3–2-5	None	Yes	Yes	Yes	No	No	Yes
6	2014	Blood	ST53	3	3–6-5	None	Yes	Yes	Yes	Yes	Yes	Yes
1	2014	Blood	ST53	3	3–6-5	None	Yes	No	Yes	Yes	Yes	Yes
1	2014	Blood	ST53	3	3–6-5	None	No	No	Yes	Yes	No	Yes
1	2014	Blood	ST53	3	3–6-5	None	No	No	Yes	Yes	Yes	No
1	2014	Blood	ST404	98	3–2-5	None	Yes	Yes	Yes	No	No	Yes
4	2016	Blood	ST53	3	3–6-5	None	Yes	Yes	Yes	Yes	Yes	Yes
1	2016	Blood	ST53	3	3–6-5	None	No	No	Yes	Yes	Yes	Yes
3	2016	Blood	ST53	3	3–6-5	None	Yes	No	Yes	Yes	Yes	Yes
1	2016	Blood	ST53	3	3–6-5	None	Yes	Yes	Yes	Yes	Yes	No
1	2016	Cerebrospinal fluid	ST53	3	3–6-5	None	Yes	Yes	Yes	Yes	Yes	Yes
7	2018	Blood	ST53	3	3–6-5	None	Yes	Yes	Yes	Yes	Yes	Yes
2	2018	Blood	ST53	3	3–6-5	None	Yes	No	Yes	Yes	Yes	Yes
1	2018	Cerebrospinal fluid	ST53	3	3–6-5	None	Yes	Yes	Yes	Yes	Yes	Yes

### Molecular species identification

WGS was performed as described by Kavalari et al [12]. The selected isolates were sequenced by paired-end Illumina sequencing, where the genomic DNA was extracted using DNeasy Blood and Tissue Kit (QIAGEN, Hilden, Germany) and fragment libraries were made by Nextera XT Kit (Illumina, Little Chesterford, UK), followed by a 250 bp paired-end sequencing (MiSeqTM; Illumina) according to the manufacturer’s instructions. The paired-end Illumina data were de novo assembled using SKESA assembler [16]. All bioinformatics were performed using the free software NCBI genome workbench (version 3.0.1, https://www.ncbi.nlm.nih.gov, accessed 14–04–2021).

### Molecular characterization of the capsular genes of the selected isolates

All 96 isolates were genetically confirmed to be *S. pneumoniae* as described by Kavalari et al. [12]. The presence/absence of a gene was based on a cut-off of 95% identity and 80% coverage as a definition of a gene detection in this study [2, 17, 18]. The isolates were serotyped using PneumoCaT (version = “1.2”) [2]. The genomic sequence data for the 96 isolates have been deposited in the ENA Genbank under project no. PRJEB42355 (https://www.ebi.ac.uk/ena/browser/view/PRJEB42355, accessed 19–04–2021).

**Fig. 1:**
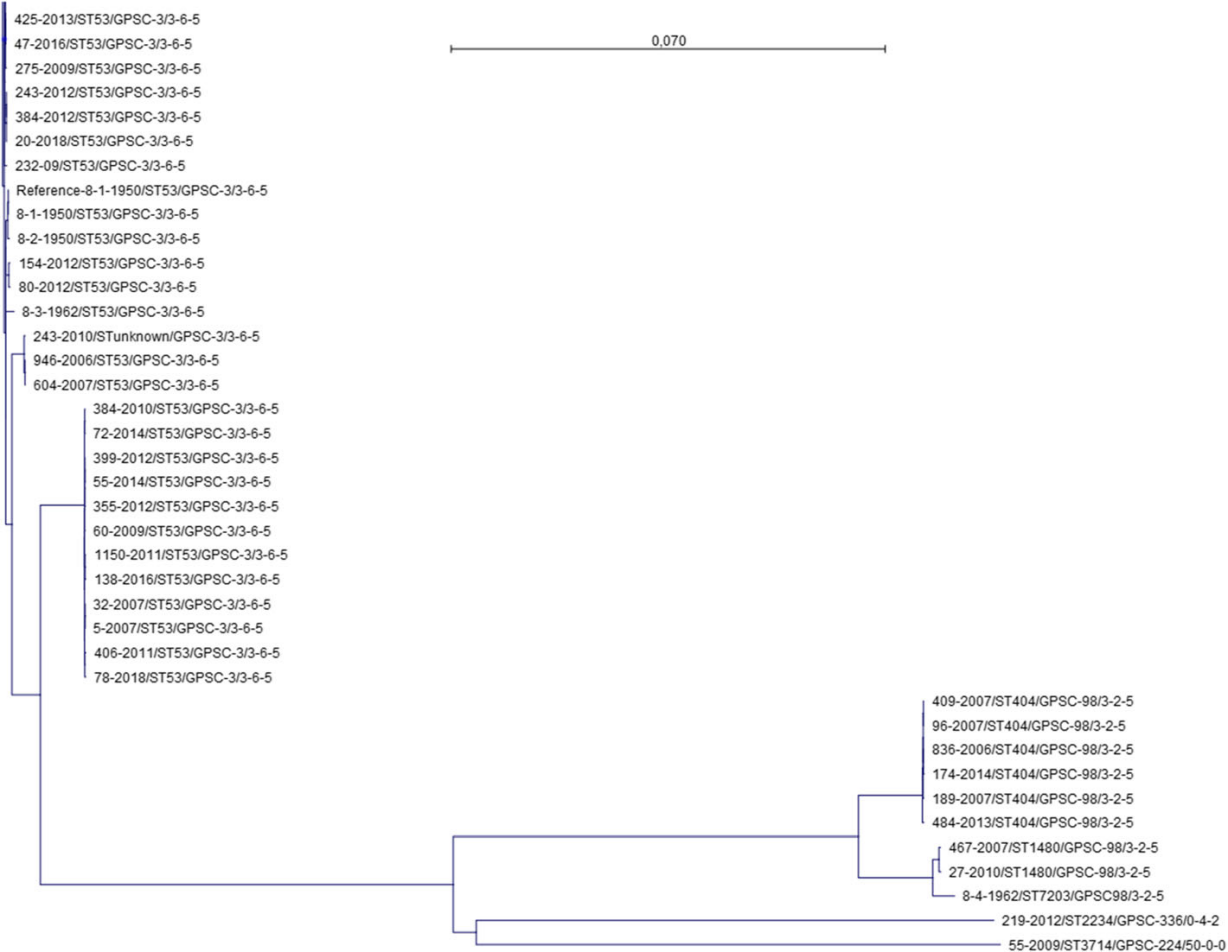
SNP alignment of 43 isolates based on their SNP site locations. *Isolate 8-1-1950 was used as a reference strain in the SNP alignment. Strain number, MLST (ST) number, GPSC number, and the PBP profile have been added to each strain. Fifty-three isolates are not presented in the figure, as they showed identical profiles to isolate 114–2014. For complete tree, see supplementary Figure 1

Species ID was confirmed using the Ribosomal MLST (rMLST) scratch database based on identification of 53 genes encoding the bacterial ribosome protein subunits (*rps* genes) (https://pubmlst.org/species-id, accessed 10–03-2021) [19]. Furthermore, the MLST profiles were determined using the PubMLST DataBase (https://pubmlst.org/spneumoniae/, accessed 10–03-2021). A phylogenetic tree based on single nucleotide polymorphism (SNP) analysis of the core genome was created and visualized in the NCBI genome workbench (version 3.0.1, https://www.ncbi.nlm.nih.gov, accessed 14–04–2021) (Fig. 1), for complete tree, see supplementary Figure 1. The SNP analysis was compared to a tree based on the rMLST species ID (https://pubmlst.org/species-id, accessed 10–03-2021) [19] (Tree not shown).

### Gene profile

The tested genes were based on selected studies:

- Presence of virulence genes *lytA* and *ply* coding for autolysin and the pore-forming toxin pneumolysin [20].
- Genes *piaA/piaB/piaC* (GenBank: AF338658.1) coding for membrane proteins and ATP-binding proteins [17, 21].
- Genes *zmpB* (from TIGR4 AE005672: 634,856-640,501) [22] and *zmpC* (from TIGR4 AE005672: 73,761-79,331) [21], which are paralogous zinc metalloproteases [23].
- The partial capsular gene *cpsA*, also known as the *wzg* gene (Genbank: AF057294:2134–2473) [24, 25].
- Genes for the pneumococcal surface proteins (*pspA*) (Genbank: AF516671) [26] and *psrp* (Genbank: A0A0H2URK1) [27].
- The gene *psaA* (Genbank: U53509.1) coding for pneumococcal surface adhesion A [20].
- Gene *rpsB* (from TIGR4 AE005672.3: 2,134,264–2,135,043) encoding for ribosomal protein S2 [21].
- Two neuraminidases *nanA/nanB* (U43526.1) expressed in *S. penumoniae* [28].
- The *Xisco* gene (*SP_1992* from TIGR4 AE005672.3: 1,895,473-1,896,138), an unique biomarker [29].
- Gene *SP2020* (from TIGR4 AE005672.3: 1,925,563-1,926,291) a putative transcriptional regulator [30].

Table 2 presents an overview of gene sequences found in the 96 isolates.

### Antibiotic susceptibility testing (phenotypic tests)

The antibiotic susceptibility testing was performed as described by Kavalari et al. [12]. Screening was done according to EUCAST (www.eucast.org) by disk diffusion using Mueller–Hinton 5% blood agar with NAD (Oxoid, Denmark) incubated in ambient air with 5% CO2 at 35 °C and oxacillin, penicillin, erythromycin and clindamycin discs (Oxoid, Denmark). Isolates showing non-susceptibility were tested using a Microbroth dilution test (Sensititre, Streptococcus species MIC Plate, STP6F, Trek Diagnostic System, USA).

### Genetic susceptibility profile

The 96 selected isolates were analyzed via Resfinder (version 3.2, https://cge.cbs.dtu.dk/services/ResFinder/, accessed 10–03-2021) to find resistance related genes such as *tetM* (FR671418), *ermB* (NCBI, FJ667782), *mefA* (NCBI, KU739790) and *mefE* (NCBI, NC_003098.1 [31]. We do not routinely perform phenotypical screening of tetracycline on the isolates.

Penicillin susceptibility of *S. pneumoniae* is associated with the penicillin binding proteins (PBP) PBP1A, PBP2B and PBP2X [32]. The 96 isolates were analyzed for their PBP signature, where the combination of the three PBP signatures determines the level of beta-lactam resistance [32].

Pathogenwatch (version 3.7.5, https://pathogen.watch/, accessed 10–03-2021) was used to determine Global Pneumococcal Sequence Clusters (GPSC), and to confirm organism, serotype, MLST and PBP.

An overview of the susceptible and related genes can be seen in Table 2.

### Ethical considerations

The data and samples from patients are collected routinely for national surveillance purposes, no ethical approval or informed consent from patients or guardians are required. Statens Serum Institut (SSI) which is under the auspices of the Danish Ministry of Health, have a general approval by the Danish Data Protection Agency (record number 2007-41-0229) (https://en.ssi.dk/research, accessed 14–04–2021, https://en.ssi.dk/about-us, accessed 14–04–2021) to publish the data. All presented data are anonymized and cannot be connected to a patient.

## Results

### Incidence of invasive pneumococcal disease in 1999–2019 due to serotype 8

The mean serotype 8 IPD incidence for most age groups in the period 2008–2010 increased compared to 1999–2007, whereas all age groups with IPD cases in 2011–2019 increased compared to 1999–2007 and 2008–2010 (Table 3). The age groups above 65 years had the highest mean incidence in 2011–2019 and the age group ‘2–4’ had the lowest one (Table 3).

**Table 3:** Statistical calculation using T-test, Fisher’s exact test and GLM on all serotype 8 IPD from 1999 to 2019

Age	1999–2007	2008–2010	2011–2019	2008–2010 versus 1999–2007 (before and after PCV7)	2011–2019 versus 2008–2010 (before and after PCV13)	2011–2019 versus 1999–2007 (before PCV7 and after PCV13)	1999–2019	2011–2019
	Mean serotype 8 IPD incidence per 100,000. 95% confidence interval of the mean incidence (lower CI; upper CI)			Two tailed Fisher’s exact test: IRR, 95% confidence interval (lower CI; upper CI).			GLM: IRR, 95% confidence interval (lower CI; upper CI).	
< 2	0.51 (0.10–0.92)	0.52 (−0.6–1.64)	1.20 (0.48–1.91)	1.02 (0.21–5.03) *P* = 1	2.33 (0.53–10.32) *P* = 0.38	2.37 (0.90–6.23) *P* = 0.11	1.07 (0.92–1.17) *P* = 0.09	1.08 (0.86–1.39) *P* = 0.49
2–4	0 (NA-NA)	0 (NA-NA)	0 (NA-NA)	NA *P* = 1	NA *P* = 1	NA *P* = 1	NA	NA
5–14	0.11 (−0.03–0.26)	0 (NA-NA)	0.12 (0.02–0.21)	0 (0-NA) *P* = 0.20	inf (NA-inf) *P* = 0.20	1.02 (0.36–2.92) *P* = 1	1.00 (0.78–1.28) *P* = 0.99	1.00 (0.42–2.40) *P* = 0.99
15–44	0.37 (0.30–0.43)	0.33 (0.13–0.53)	0.69 (0.42–0.95)	0.89 (0.55–1.45) *P* = 0.72	**2.11 (1.33–3.35)** ***P*** **< 0.001**	**1.89 (1.42–2.52)** ***P*** **< 0.001**	1.07 (0.96–1.92) *P* = 0.23	1.17 (0.86–1.67) *P* = 0.33
45–64	1.26 (1.06–1.46)	1.39 (0.90–1.88)	2.71 (2.04–3.37)	1.10 (0.82–1.47) *P* = 0.54	**1.95 (1.49–2.55)** ***P*** **< 0.001**	**2.14 (1.78–2.58)** ***P*** **< 0.001**	**1.06 (1.01–1.12)** ***P*** **= 0.03**	1.09 (0.93–1.28) *P* = 0.28
65–74	2.98 (2.46–3.49)	2.70 (1.22–4.17)	6.41 (4.27–8.56)	0.92 (0.64–1.30) *P* = 0.66	**2.40 (1.74–3.30)** ***P*** **< 0.001**	**2.20 (1.78–2.71)** ***P*** **< 0.001**	**1.04 (1.01–1.08)** ***P*** **= 0.02**	**1.13 (1.02–1.26)** ***P*** **= 0.02**
75–84	3.59 (2.74–4.44)	5.16 (1.53–8.80)	7.62 (5.88–9.36)	1.44 (1.00–2.07) *P* = 0.06	**1.49 (1.08–2.07)** ***P*** **= 0.02**	**2.15 (1.68–2.74)** ***P*** **< 0.001**	**1.04 (1.01–1.08)** ***P*** **= 0.01**	1.02 (0.93–1.11) *P* = 0.73
85+	3.07 (2.29–3.86)	6.10 (−3.47–15.6)	11.34 (7.42–15.26)	**1.97 (1.11–3.50)** ***P*** **= 0.022**	**1.89 (1.18–3.03)** ***P*** **= 0.0073**	**3.72 (2.47–5.61)** ***P*** **< 0.001**	**1.1 (1.07–1.13)** ***P*** **< 0.001**	**1.14 (1.06–1.23)** ***P*** **< 0.001**
Total	0.99 (0.89–1.09)	1.14 (0.91–1.38)	2.40 (1.73–3.07)	1.15 (0.97–1.36) *P* = 0.10	**2.11 (1.81–2.46)** ***P*** **< 0.001**	**2.43 (2.18–2.70)** ***P*** **< 0.001**	1.08 (1.02–1.14) *P* = 0.015	1.12 (0.95–1.33) *P* = 0.20

*GLM* Generalised linear model, *IRR* incidence risk ratio, *NA* no value, Total = all age groups, Bold = statistical significance

The effect of PCV7 on serotype 8 IPD (introduced gradually in 2007) using a two-tailed Fisher’s exact test by comparing period 1999–2007 to period 2008–2010 showed a significant difference in serotype 8 IPD incidence for the age group ‘85+’ (IRR = 1.97, CI: 1.11–3.50, *P* = 0.02) (Table 3).

The effect of PCV13 on serotype 8 IPD (introduced gradually in 2010) using a two-tailed Fisher’s exact test by comparing period 2008–2010 to period 2011–2019 showed a significant increase in the incidence of serotype 8 IPD for all groups above the age of 15 (Table 3).

**Fig. 2:**
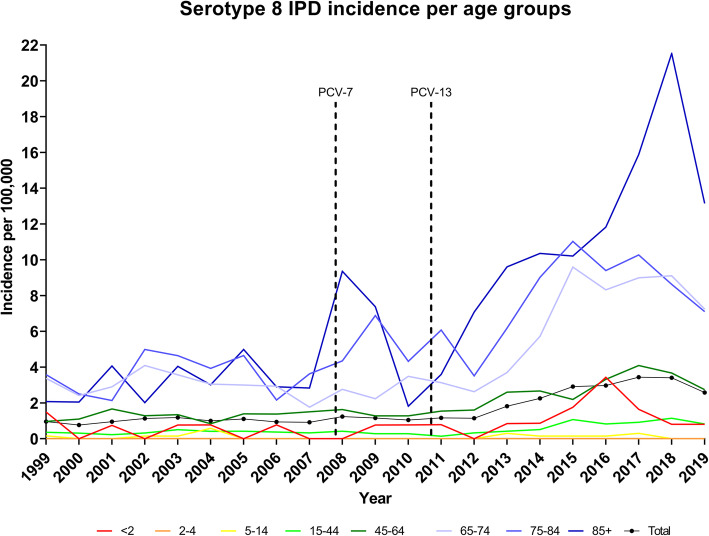
Incidence of serotype 8 IPD per 100,000. Arrows indicate when PCV7 and PCV13 were introduced. ‘Total’ includes all age groups

The effect of the PCV vaccines against serotype 8 IPD using the GLM statistic, the serotype 8 IPD incidence in the age groups ‘65–74’ and ‘85+’ (*P* = 0.02 and *P* = 0.001, respectively) increased significantly with an average of 13–14% per year in the period from 2011 to 2019. The serotype 8 IPD incidence peaked in 2015–2018 and decreased for the majority of age groups in 2019 (Fig. 2).

### MLST

Seven different MLST profiles were detected among the 96 isolates of serotype 8, of which 84 (87.5%) were ST53, six ST404, two ST1480, one ST7203, one ST3714, one ST2234, and one had an unknown ST profile. Four different GPSC were detected, of which 85 were GPSC3 (consisting of ST53 and the unknown ST), nine GPSC98 (consisting of ST404, ST1480 and ST7203), one GPSC224 (consisting of ST3714) and one GPSC336 (consisting of ST2234) (Fig. 1, Table 2). It was not possible to detect any clustering. ST53 was detected in isolates from all parts of the country, ST404 was detected in isolates from four different hospitals from both Jutland and Zealand, and ST1480 were detected in samples from two different hospitals representing Jutland and Zealand.

### Comparison of phenotypic and genotypic susceptibility profiles

All isolates were genetically identified as serotype 8, confirming the detected phenotype. All of the 92 recent isolates with available antimicrobial susceptibility profiles were phenotypically susceptible to penicillin, erythromycin, and clindamycin. Four different PBP signatures were detected of which 85 isolates were ‘3–6-5’, nine were ‘3–2-5’, one ‘50–0-0’, and one was ‘0–4-2’ (Fig. 1, Table 2).

When performing a ResFinder search, three isolates showed genes with 99% identity to tetracycline resistance genes. The isolates were of sequence types ST53 and the clonally related new ST type (Fig. 1 and Table 2). The estimated tetracycline resistance is 3.13% of the total 96 isolates based on WGS.

### Comparison of phylogenetic trees

A correlation of isolates GPSC, MLST and PBP was demonstrated in the SNP phylogenetic tree when isolates in the same GPSC clustered together and grouped according to MLST and PBP (Fig. 1). All ST53 isolates were arranged close to the root isolate (‘8-1-1950’), with two clades of twelve and three ST53 isolates in a separate clade further from the root. The clade with three isolates had genes for tetracycline resistance (isolates 243–2010, 604–2007, 946–2006). Comparison of the SNP tree with an rMLST-based tree from PubMLST showed identical branches and separation of isolates (data not shown).

### Characterization of selected pneumococcal genes

The genes *lytA, ply, xisco, rpsB, pspA,* and *psaA* were detected in all 96 pneumococcal serotype 8 isolates, the genes *sp2020, piaA/piaB/piaC*, *zmpC,* and *nanA/nanB* were detected in the majority of isolates, and the genes *zmpB, cpsA* and *psrP* were absent in all isolates (Table 2).

*PiaA/piaB/piaC* were present in all of the isolates before PCV13 introduction and absent in 12.5% of the isolates after the introduction of PCV13 (Table 2). Only ST53 and the clonally related isolates carried the *zmpC* gene, while it was absent in all other isolates.

## Discussion

Introduction of the PCV vaccines has reduced IPD in children and other age groups. However, the introduction of the PCV vaccines has also contributed to an increase in the proportion of the non-vaccine serotypes such as serotype 8 in countries like Denmark, the United Kingdom, Spain, and other European countries [6], showing a post-vaccination serotype replacement [5, 6, 33, 34].

The serotype 8 IPD incidence in Denmark increased after the PCV13 introduction, predominantly affecting the age groups above 65 years, and a significant increase was observed for the age group 85+ after the introduction of PCV7; the serotype 8 IPD incidence increased in 2008–2009 and then decreased to pre-PCV7 levels in 2010 (Fig. 2, Table 3). The large increase of serotype 8 in Denmark (Fig. 2, Tables 1 and 3) after the introduction of PCV13 was not foreseen in any Danish published IPD and pneumococcal carriage data up to December 2013 [4, 13, 35]. It was observed that around 80% of IPD cases in 2012–2013 were caused by non–vaccine serotypes (8, 10A/B, 12F, 15B/C, 20, 22F, 33F, 38, 23B, 24F), with no clear predominance of any specific serotype [4]. In 2014, Danish IPD data on non-vaccine serotypes indicated the dominance of serotype 8, although at that time it was not clear that serotype 8 would continue to be the leading cause of IPD in Denmark (Table 1) [5]. Neither did Danish carriage studies in children below 5 years of age in 2000 [35] and below 2 years of age in 2014 to 2016 [36] show any indication of high carriage of serotype 8, which could explain the transmission to the elderly. Similar carriage data on serotype 8 in children below 5 years of age showing limited carriage have been observed in other countries [37]. It has furthermore been found that there is a limitation in using carriage data from children to forecast changes in general IPD epidemiology, and that serotype 8 is a possible example of a serotype transmitted directly among older age groups [38]. This observation is supported by studies from the UK performed on other age groups than children, in which they observed serotype 8 carriage [37, 39]. In Denmark, no carriage studies on other age groups than children have been performed, which suggests a direct transmission among other age groups [36].

The current Danish pneumococcal data are not able to provide an explanation or warning of the present dominance of serotype 8 [4, 5, 13, 35, 36]. Moreover, the epidemiological data does not provide an explanation for the dominance of serotype 8 IPD cases observed in Denmark.

At present only the pneumococcal polysaccharide vaccine (PPV23) includes serotype 8, which has shown a significant vaccine efficacy against serotype 8, although the protection is of limited duration [40]. The duration of protection can explain the limited effect of PPV23 in England against serotype 8 IPD despite a national PPV23 immunization program for the age group of 65+ since 2003 [40, 41]. The serotype 8 IPD in Denmark predominantly affects the age groups above 65 years (Fig. 2, Tables 1 and 3), and it will be important to monitor the serotype 8 IPD incidence with the introduction of PPV23 into a vaccination program for risk groups and the elderly 65+ [42].

Serotype 8 is often observed to be susceptible to antimicrobial drugs [8]. Spain has, however, seen an emergence and spread of *S. pneumoniae* serotype 8 ST63, a multidrug resistant clone resistant to erythromycin, clindamycin, tetracycline, and ciprofloxacin [8].

In Denmark we have not observed any occurrence of non-susceptible serotype 8 isolates (DANMAP, https://www.danmap.org/, accessed 10–03-2021), and the post PCV13 increase in serotype 8 incidence has not shown any changes in the susceptibility of serotype 8 isolates. The PBP profiles of the sequence isolates in this study corresponded well with the predicted PBP profile and the phenotypic susceptibility testing (Fig. 1) [12, 32].

The *S. pneumoniae* serotype 8 MLST type is ST53 belonging to cluster GPSC3 [43–46], constituting 80% of the sequenced isolates in this study. The ST53 clone was found to be dominant both before and after the introduction of PCV7 and PCV13 (Table 2). The increase in serotype 8 can, therefore, not be related to changes in serotype 8 clones. Other serotype 8 MLST types observed in this study, such as ST404 and ST1480, have been reported in other European countries, Brazil, and The UK [45, 47–51], while MLST types ST3714 and ST2234 have only been observed in Denmark, Sweden, Turkey, Belarus, the UK, Saudi Arabia, and Kenya (PubMLST DataBase, https://pubmlst.org/spneumoniae/, accessed 10–03-2021). The historical isolate 8-4-1962 (ST7203) was related to clone ST404 and was in the same GPSC98 cluster. An unknown ST type was detected in isolate 243–2010, which had six of seven identical allelic variants with ST53 and was in the same GPSC3 cluster (Fig. 1). Overall, all MLST types in this study were known as susceptible clones, although three isolates showed the presence of the *tet(M)* gene (Table 2).

The SNP phylogenetic tree showed that it was not possible to see any clades of isolates segregated by the year before and after the PCV introduction, indicating that it might not be a gene mutation causing the serotype 8 increase (Fig. 1). The tree illustrates two clades of twelve and three ST53 isolates, respectively, that were separated from the majority of ST53 isolates. The differentiation of the clades could, however, not be linked to the year of isolation. We do not know the basis of the difference for the twelve isolates based on the genes selected in this study, and further gene analysis needs to be performed to reveal which genes were responsible for the discrepancy. The clade of three isolates showed molecular tetracycline resistance, differentiating them from the majority of the ST53 isolates.

Comparing the SNP tree with a tree based on the 53 rMLST genes from PubMLST species identification showed nearly identical branches, although the SNP tree showed more details in the branches, as the clade with the three isolates containing the *tet(M)* gene was not present in the rMLST tree (data not shown). In general, the authors found that the species ID identification using PubMLST rMLST was easy to use; however, it did not provide any additional information on the cause for the increase in serotype 8.

Evaluation of species-specific genes described in various studies [12, 20, 30, 52] did not show a clear presence/absence of genes defined by the PCV introduction (Table 2). The generally used *lytA* gene and other genes suggested for species identification of *S. pneumoniae* were detected in all our isolates (Table 2) similar to our previous observations [12]. Some genes were not observed in all our isolates; *SP2020* [30] was not found in two of our isolates (Table 2). The *zmpC* gene was present in all ST53 isolates and in the clonally related isolate, while it was absent in all other ST types, which is consistent with observations from previous studies [22]. However, interestingly the *zmpC* gene has been described to suppress *S. pneumoniae* virulence in experimental models of pneumococcal meningitis [21]. In this study, specific meningitis data are too limited to evaluate the effect on the number of meningitis cases; however, the *zmpC* gene was found in isolates from cerebrospinal fluid and did not seem to be linked to reduced invasiveness of serotype 8 (Table 2). The genes *piaA/piaB/piaC* were present in all isolates before the introduction of PCV13. However, they were lacking in 12.5% of the isolates (8 isolates) after the introduction. Although the absence of the genes *piaA/piaB/piaC* first appeared after the PCV13 introduction, it does not explain the increase in serotype 8, as only a limited number of isolates lacked the genes (Table 2).

Interestingly, the *SP2020* or *piaB* gene in combination with the *lytA* gene has been suggested for the detection of pneumococcal pure cultures or swab samples [21, 30]. However, when analyzing the 96 isolates in this study, we observed isolates which did not include the *SP2020* or *piaB* gene (Table 2). It is therefore questionable how favorable these genes are compared to the use of the *ply* gene for detection of Danish pneumococcal isolates. All isolates in this study (Table 2) and the study by Kavalari et al [12] showed the presence of the *ply* gene. It has furthermore been described that the *piaB* gene only lacks in non-typeable pneumococci [21]. In this study, however, the *piaB* gene was not found to be unique for the invasive capsulated isolates, as 7 isolates lacked the gene (Table 2).

A limitation of the study includes that not all serotype 8 isolates were sequenced with the caveat that we might not detect possible mini-outbreaks of specific clones as a possible explanation for the observed serotype 8 replacement in Denmark. However, the number of isolates was sufficient enough to find interesting sequence results (e.g. isolates not possessing the *SP2020* or *piaB* genes (Table 2).

## Conclusion

In conclusion, with the introduction of PCV13 in the child vaccination program in Denmark, a significant (*P* < 0.05) increase was observed in the non-PCV serotype 8 IPD incidence for the age groups above 65 years, demonstrating serotype replacement in Denmark. No reason was found for the successful replacement of serotype 8 based on the Danish epidemiological studies. Furthermore, the increase in serotype 8 was not followed by an increase in non-susceptible serotype 8 isolates or by a change in clones, as the majority of molecularly characterized isolates belonged to the ST53 clone. Analysis of potential changes in the clonal distribution, molecularly susceptible related genes, and species-specific genes pre- and post-PCV vaccination did not show any changes which could be related to the PCV introduction in Denmark. Therefore, future studies still need to identify a possible marker for why serotype 8 is so successful in replacing the PCV included serotypes in Denmark, and thereby possibly improve the prediction of the next non-PCV serotype causing high incidence of IPD in Denmark.

## Supplementary Information

**Additional file 1.**

## Data Availability

The datasets used and/or analysed during the current study are available from the corresponding author on reasonable request. The genomic sequence data for the 96 isolates have been deposited in the ENA Genbank under project no. PRJEB42355 (https://urldefense.proofpoint.com/v2/url?u=https-3A__www.ebi.ac.uk_ena_browser_view_PRJEB42355&d=DwIGaQ&c=vh6FgFnduejNhPPD0fl_yRaSfZy8CWbWnIf4XJhSqx8&r=yAMxyalBUU6FWS_X92e5RF0qgu27J-fAum9UIH64Ji9P7WvInM6e9epg0HCgDPW7&m=MdhWCdzhjtVxwm-mDbI3863amjzJ3nOl0U2rUseOpT0&s=Lu1fuwzMM9TAh18D0ZPl86m3jMcHOlP15GdpRPJrLLU&e=, accessed 19-04-2021).

## Abbreviations

IPD: Invasive pneumococcal disease
PCV: Pneumococcal conjugated vaccine
MLST: Multi locus sequence type
WGS: Whole-genome sequencing
IRR: Incidence rate ratio
CI: Confidence interval
rMLST: Ribosomal MLST
SNP: Single nucleotide polymorphism
PBP: Penicillin binding protein
GPSC: Global pneumococcal sequence cluster
