# Supplementary figures and images for: Molecular characterization and epidemiology of Streptococcus pneumoniae serotype 8 in Denmark
Source: BMC Infect Dis. 2021 May 5;21:421. doi: 10.1186/s12879-021-06103-w (PMC8097992; doi:10.1186/s12879-021-06103-w)

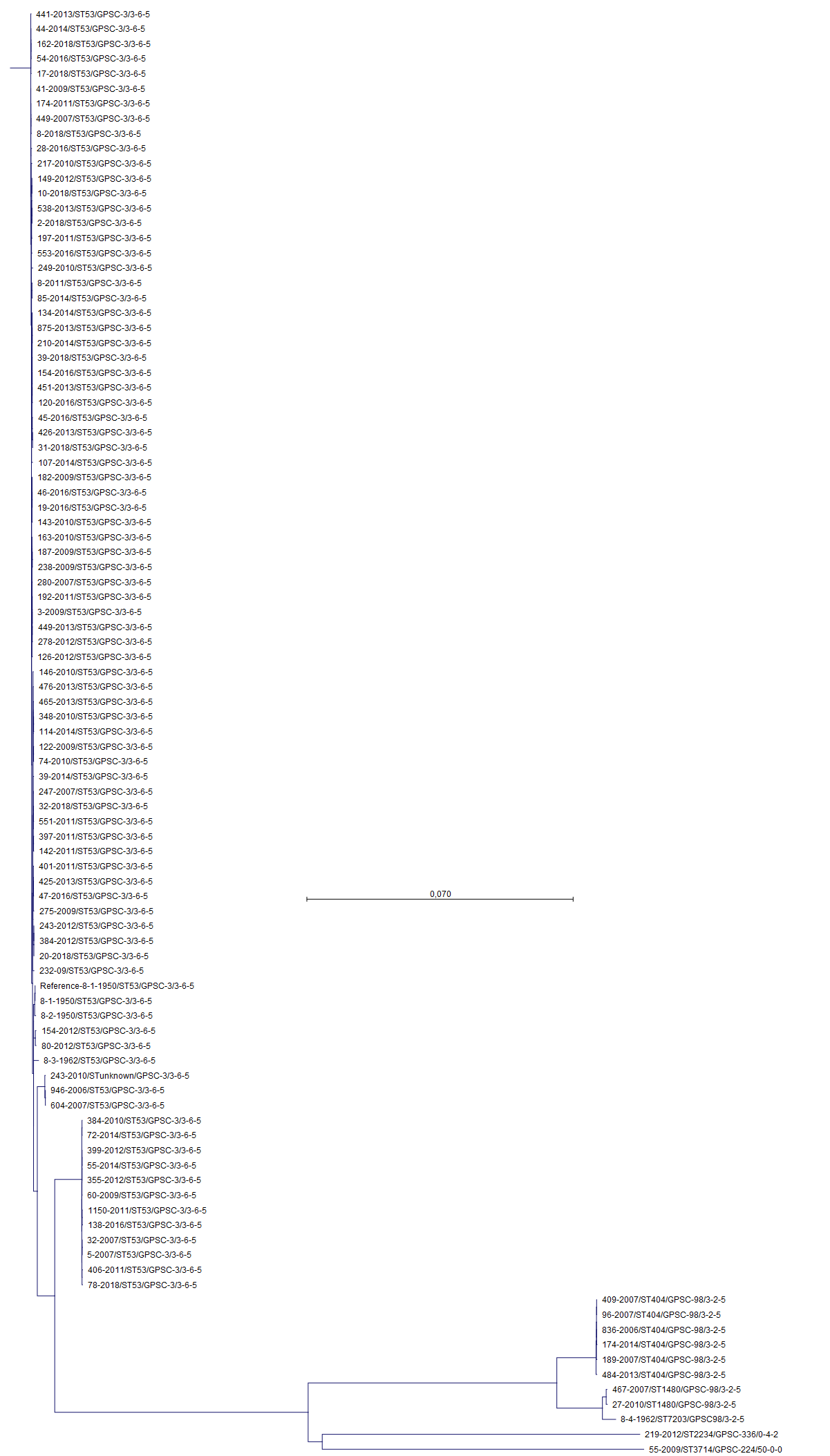

Supplement: Supplementary file 1 — Additional file 1. [file 12879_2021_6103_MOESM1_ESM.tif]
